# Poor oral health and risk of incident myocardial infarction: A prospective cohort study of Swedish adults, 1973–2012

**DOI:** 10.1038/s41598-018-29697-9

**Published:** 2018-07-31

**Authors:** Katherine Wilson, Zhiwei Liu, Jiaqi Huang, Ann Roosaar, Tony Axéll, Weimin Ye

**Affiliations:** 1 Department of Medical Epidemiology and Biostatistics, Karolinska Institutet, Stockholm, Sweden; 2 Department of Dental Medicine, Karolinska Institutet, Stockholm, Sweden; 3 Maxillofacial Unit, Halmstad Hospital Halland, 30185 Halmstad, Sweden

## Abstract

Previous studies provide conflicting evidence as to whether there is an association between poor oral health and an increased risk of myocardial infarction. The aim of the study was to deepen knowledge of the association between oral health and myocardial infarction risk using a large (n = 20,133), prospective, and population-based cohort in Uppsala, Sweden. Oral health was determined during a clinical dental examination at entry into the cohort in 1973/74. Individuals were followed through linkage with the Swedish National Patient Register, Cause of Death Register and Emigration Register. Cox proportional hazards regression models were used to estimate hazard ratios (HRs) for total, non-fatal and fatal myocardial infarction events. Increased risks of total, non-fatal and fatal myocardial infarction events among individuals with fewer reference teeth at examination, more dental plaque and a borderline significant increased risk among individuals with oral lesions were observed. Adjustment for multiple potential confounding factors did not change the results appreciably. However, the observed HRs generally decreased towards one when the analysis was confined to non-tobacco users only. The results from this study indicate that poor oral health is associated with a slightly increased risk of myocardial infarction; however, these results may be partly explained by residual confounding.

## Introduction

Cardiovascular disease (CVD) is one of the leading causes of death and morbidity globally, with an estimated 17.5 million deaths due to CVD in 2012^1^ and 295 million disability-adjusted life years (DALYs) in 2010^2^. There are many identified risk factors for CVD including tobacco smoking^3^, high alcohol consumption^4^, sedentary lifestyle^5^, high blood pressure^6^ and genetic predisposition^7^. Since the 1990s, poor oral health has also been identified as a potential risk factor for CVD^8^. Poor oral health is considered generally to be one of the most prevalent diseases globally, with 32% of individuals worldwide aged 65 years or older estimated to be edentulous (having no natural teeth)^9^. Furthermore, a study in the United States observed a 28% prevalence of oral mucosal lesions in a nationally representative sample of individuals^10^.

Previous studies have observed an increased risk of myocardial infarction (MI) with various measures of oral health including tooth loss^11^, periodontal diseases^12^, and dental plaque^13^. In contrast, others have observed null association and suggested that the reported associations are a result of uncontrolled confounding^14–16^. Thus, there is still debate regarding the association between oral health and MI, and whether any association is causal.

To investigate the association between oral health and the risk of MI events, we examined the hazards of total, non-fatal and fatal incident MI events in relation to the number of teeth, presence of dental plaque and presence of oral lesions in a large, prospective, and population-based cohort in Uppsala, Sweden.

## Materials and Methods

### Study population

**Figure 1:**
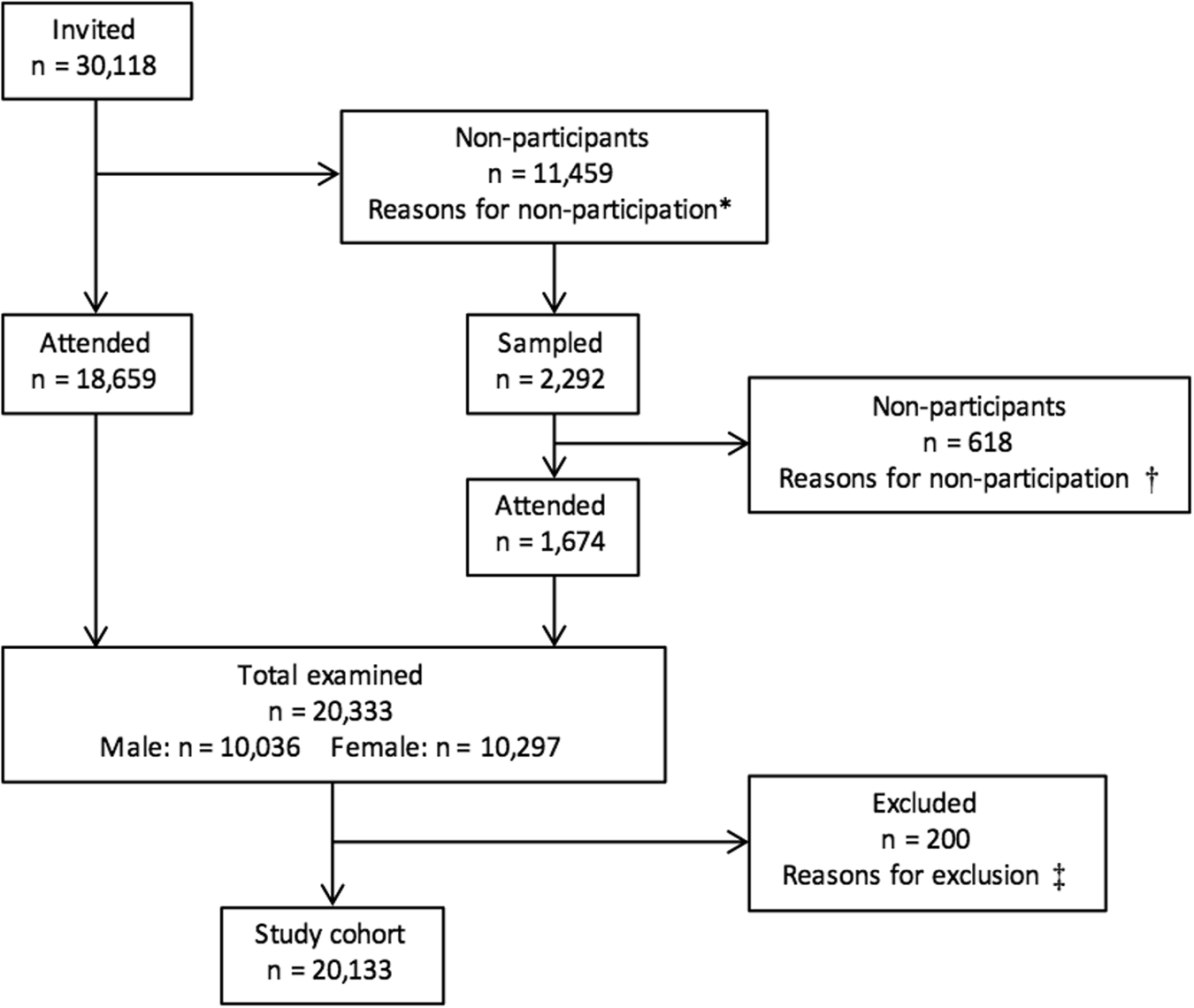
Flow diagram for participant recruitment into the cohort in 1973/74. ^*^Reasons for non-participation in first round of recruitment (sample of non-participants, n = 2,382): Recently participated in another health examination (n = 371), work (n = 328), temporarily away (n = 281), no contact (n = 321), change of residence (n = 224), illness at home (n = 152), no remembrance of reason (n = 149), refusal (n = 149), hospitalised (n = 115), did not receive parcel (n = 64), forgot to come (n = 48), old age (n = 35), recently dead (n = 28), long distance to examination local (n = 24), fear of physicians and/or dentists (n = 20), dislike of mass investigations (n = 18), pregnancy (n = 10), other reasons (n = 45). ^†^Reasons for non-participation in second round of recruitment: Change of residence (n = 308), no contact (n = 97), recently dead (n = 84), refusal (n = 84), temporarily away (n = 11), hospitalised (n = 10), recently participated in another health examination (n = 5), illness at home (n = 4), dislike of mass investigations (n = 3), fear of physicians and/or dentists (n = 1), work (n = 1), other reasons (n = 10). ^‡^Reasons for exclusion: MI event prior to entry into cohort (n = 79), data missing/unreadable (n = 60), no birth date (n = 23), surveyed two times (n = 15), changed personal identification number between 1973 and 1998 (n = 14), incorrect personal identification number (n = 8), duplicated study ID (n = 1).

All individuals aged 15 years or older during the year of examination and registered in the National Civil Register as living in the municipalities of Enköping or Håbo, in Uppsala County in Sweden were invited to take part in the study (n = 30,118). After two rounds of recruitment, a total of 20,333 individuals received a clinical dental examination in 1973/74, resulting in a response rate of 68 percent (Fig. 1). The original selection of participants and data collection was for the purpose of studying the prevalence of oral mucosal lesions in Sweden^17^. Individuals were excluded if they had a MI event prior to entry into the cohort (n = 79). Entry into the cohort was set to the 15^th^ day of the month of dental examination as only a record of the month and year was available. Ethical approval has been granted by the ethics committee of the Medical Faculty at Uppsala Universitet (DNR: 82/93) and the Regional Ethics Vetting Board in Stockholm, Sweden (DNR: 2014/671–31/4). Informed consent was obtained from all participants. All methods were performed in accordance with the relevant guidelines and regulations.

### Oral health status

All dental examinations were conducted at entry into the cohort by the same dentist (T. Axéll)^17^. Three measurements indicating the status of oral health are considered: number of teeth, severity of dental plaque and the presence of oral lesions.

Number of teeth was assessed by examination of six reference teeth (tooth numbers 16, 21, 24, 36, 41, 44 using the Ramfjord teeth index)^18^. Number of teeth present was grouped in the analysis to 6, 4–5, 2–3 and 0–1, as a measure of general decreasing oral health. We assume that because the six reference teeth are spread throughout the mouth, that individuals with all six of the reference teeth present during examination, are more likely to have more teeth overall, compared to those with fewer reference teeth present. Individuals with 6 teeth were used as the reference group^19^.

Dental plaque was examined on each of the six reference teeth. Each tooth was assigned a score between 0 and 3, with 0 indicating no dental plaque, 1 indicating ‘soft debris covering not more than one third of the tooth surface, or presence of extrinsic stains without other debris regardless of the surface area covered’, 2 indicating ‘soft debris covering more than one third, but not more than two thirds, of the exposed tooth surface’, and 3 indicating ‘soft debris covering more than two thirds of the exposed tooth surface’. The dental plaque score was calculated from examination of the six reference teeth, as the mean score for the number of teeth examined. To increase the accuracy of this variable, a dental plaque score was only calculated for individuals with two or more of the reference teeth present (n = 15,509). The reference group was those with no dental plaque.

Oral lesions present during the dental examination were recorded and history of herpes and apthae lesions was reported by the study subjects, as these lesions can be recurrent. Oral lesions are grouped according to their aetiology or position in the mouth: *Candida*-related lesions, denture-related lesions and tongue lesions. The *Candida*-related lesions group contains the following conditions: pseudomembranous candidiasis, chronic candidosis, angular cheilitis, atrophic and nodular leukoplakia, median type of atrophy of tongue papillae and glossitis, and unspecified. Denture-related lesions include: denture stomatitis (localised, generalised and papillomatous), denture hyperplasia, traumatic ulcer and flabby ridges. Tongue lesions include: lingua geographica, geographic stomatitis, lingua fissurata, plicated tongue, atrophy of tongue papillae, hairy tongue, coated tongue, median rhomboid glossitis and glossitis unspecific. These groupings were chosen after consultation with the dentists involved in the original data collection. Individuals with none of the above-mentioned oral lesions were used as the reference group.

### Covariates

In the analysis, age, sex, tobacco use, alcohol consumption, calendar period and socioeconomic status (SES) were considered potential confounders. Age at entry into the cohort was used as a continuous variable in the statistical analysis, and sex was categorised as male or female. Tobacco use and alcohol consumption were determined from the self-administered questionnaire completed at the time of dental examination. Tobacco use includes both questions on ever and current smoking and moist snuff (snus) use, and was grouped into one of the categories: uses neither, smoker only, snus user only, uses both. For the non-tobacco user analysis, this subgroup contains only individuals who used neither. Alcohol consumption was categorised into: no/low (consumption less than once a week) and moderate/high (consumption once a week or more). A measurement of socioeconomic status was estimated based on the area of residence of the individual at study entry (city, small town, rural), determined from the National Civil Register.

### Follow-up and case identification

Members of the cohort were followed prospectively for incident MI through linkage with the Swedish Patient (inpatient and outpatient) Register, as well as the National Cause of Death Register, using an individual’s unique personal identity number (personnummer). Individuals (n = 20,133) were followed until MI event, death due to another cause, migration out of Sweden or to a county in Sweden not completely covered by the Inpatient Register at the time of move, or the end of follow-up on 31^st^ December 2012, whichever came first. MI events were grouped as non-fatal or fatal, and total (both non-fatal and fatal). Including separate subgroups of fatal and non-fatal MI events allows comparison with previous studies which have only measured one of these subgroups as the outcome.

Cases of MI were identified using the International Classification of Disease (ICD), seventh (years 1964–68), eighth (1969–86), ninth (1987–96) and tenth (1997-present) revisions^20^. ICD-7 codes 420.10 and 420.17, ICD-8/9 code 410 and ICD-10 codes I21 and I22 were used to identify cases of MI. To ensure high specificity of the outcome, we only considered MI recorded as the main diagnosis in the Patient Register or as the underlying cause of death in the Cause of Death Register; this approach has been used previously^21,22^. In the Patient Register, a missing primary diagnosis is present in 0.8% of somatic care and 2.4% of geriatric care^23^. MI diagnosis in the Inpatient Register has a positive predictive value (PPV) of 98–100%^23,24^, and a sensitivity of 77–92%^23,25,26^.

The Inpatient Register was established in 1964/65 in the Uppsala region in Sweden. Since 1987 the Inpatient Register has had a coverage of almost 100%, however, the Outpatient Register was established in 2001 and currently has a coverage of around 80%^23^. This is not likely to be a problem for this study as most MI events would involve hospitalisation^27^. The National Cause of Death Register was established in 1952. In this study, fatal MI events were defined as death within 28 days of first hospital admission or outpatient contact with a main discharge diagnosis of MI and with an underlying cause of death of MI, or only a record of underlying cause of death of MI in the Cause of Death Register.

### Statistical analysis

Age-standardised incidence rates of MI were calculated, standardised to the age distribution of person-years experienced by all members of the cohort (n = 20,133), using 5-year intervals. Cox proportional hazards regression models were used to calculate hazard ratios (HRs) and 95% confidence intervals (CIs) adjusting for potential confounding variables, sex, smoking and snus use, alcohol consumption and area of residence. Attained age was used as the time scale in the Cox proportional hazards models to minimise confounding by age^28^. The models were further stratified by attained calendar period in 5-year categories (1973–1977, 1978–1982, 1983–1987, 1988–1992, 1993–1997, 1998–2002, 2003–2007, 2008–2012). Individuals who had missing values for any of the variables were excluded from the Cox proportional hazards models. Ties in the Cox models were handled according to the Breslow method. Proportional hazards assumptions were checked using Schoenfeld residuals^29^. The models were stratified by covariates which violated the proportional hazards assumption, and a multiplicative interaction term between an oral health variable and attained age was added to the model if that oral health variable violated the assumption. Analysis was also conducted separately in males and females, and in non-tobacco users in order to reduce residual confounding by smoking and snus use, as suggested in previous studies^30^. Statistical analyses were performed using SAS software version 9.4 (SAS Institute Inc., Cary, NC, USA) and Stata software version 13 (StataCorp. 2013. *Stata Statistical Software: Release 13*. College Station, TX, USA: StataCorp LP.). A two-sided *P*-value < 0.05 was considered statistically significant.

### Data availability

The original data of the Uppsala Dental health check-up cohort and the linkage data from various health and demographic registers (de-identified) are currently stored in the Department of Medical Epidemiology and Biostatistics, Karolinska Institutet. Access to this database will be granted, on condition that researchers have appropriate ethical permission and sign the appropriate Material Transfer Agreement form.

## Results

After a median follow-up time of 33.8 years, corresponding to 548,210 person-years, 2,971 cases of MI (2,034 non-fatal and 937 fatal events) were diagnosed among 20,133 individuals in the cohort. At entry into the cohort, 4,624 individuals (23%) had only 0 or 1 of the six reference teeth remaining and 2,639 (13%) had high levels of dental plaque (Table 1); 1,179 (6%) had *Candida*-related lesions present, 4,256 (21%) had denture-related lesions and 3,061 (15%) had tongue lesions (data not shown).

Number of teeth, dental plaque and presence of oral lesions were correlated to the age of the individuals at cohort entry. Older participants were more likely to have fewer teeth, a higher level of dental plaque, and presence of oral mucosal lesions (Table 1).

**Table 1:** Baseline characteristics of cohort members (n = 20,133), number (N) and percentage (%).

	Number of teeth^*^				Dental plaque			
	0–1 N(%)	2–3 N(%)	4–5 N(%)	6 N(%)	Not accurate^†^ N (%)	No dental plaque N (%)	Low dental plaque N (%)	High dental plaque N (%)
Total number	4,624 (23.0)	2,235 (11.1)	3,752 (18.6)	9,522 (47.3)	4,624 (23.0)	3,224 (16.0)	9,646 (47.9)	2,639 (13.1)
**Age, years**								
Median	63.9	55.0	45.4	28.9	63.9	34.9	34.0	40.6
Percentile (25^th^−75^th^)	54.6–72.4	45.9–63.6	35.6–55.2	22.2–37.2	54.6–72.4	26.3–47.7	25.6–47.1	28.3–56.8
**Sex**								
Male	1,970 (42.6)	1,139 (51.0)	1,876 (50.0)	4,930 (51.7)	1,970 (42.6)	1,082 (33.6)	4,949 (51.3)	1,914 (72.5)
Female	2,654 (57.4)	1,096 (49.0)	1,876 (50.0)	4,592 (48.2)	2,654 (57.4)	2,142 (66.4)	4,697 (48.7)	725 (27.5)
**Smoking and snus use**								
Uses neither	2,429 (52.5)	1,046 (46.8)	1,685 (44.9)	4,203 (44.1)	2,429 (52.5)	1,572(48.8)	4,386 (45.5)	976 (37.0)
Smoker only	1,820 (39.4)	1,022 (45.8)	1,795 (47.8)	4,574 (48.0)	1,820 (39.4)	1,487 (46.1)	4,530 (47.0)	1,374 (52.1)
Snus user only	245 (5.3)	104 (4.7)	143 (3.8)	374 (3.9)	245 (5.3)	83 (2.6)	382 (4.0)	156 (5.9)
Uses both	130 (2.8)	62 (2.8)	129 (3.4)	371 (3.9)	130 (2.8)	81 (2.5)	348 (3.6)	133 (5.0)
**Alcohol consumption**								
No/Low	1,828 (39.6)	594 (26.6)	735 (19.6)	1,374 (14.4)	1,828 (39.5)	514 (15.9)	1,654 (17.2)	535 (20.3)
Moderate/High	2,791 (60.4)	1,640 (73.4)	3,015 (80.4)	8,148 (85.6)	2,791 (60.4)	2,709 (84.0)	7,991 (82.8)	2,103 (79.7)
**Area of residence in 1973/74**								
Small town	545 (11.8)	285 (12.8)	489 (13.0)	1,790 (18.8)	545 (11.8)	341 (10.6)	1,600 (16.6)	623 (23.6)
Rural	1,857 (40.1)	829 (37.1)	1,308 (34.9)	2,932 (30.8)	1,857 (40.2)	1,021 (31.7)	3,140 (32.6)	908 (34.4)
City	2,222 (48.1)	1,121 (50.2)	1,955 (52.1)	4,800 (50.4)	2,222 (48.1)	1,862 (57.8)	4,906 (50.9)	1,108 (42.0)

^*^Number of teeth was assessed by examination of six reference teeth (tooth numbers 16, 21, 24, 36, 41, 44 using the Ramfjord teeth index). ^†^The measurement of dental plaque for individuals with only 0 or 1 of the reference teeth present was considered to not be accurate.

### Age-standardised incidence rates (aSIR)

There was an increasing aSIR of MI events (total), with decreasing number of the reference teeth present, 410, 524, 639, 994 per 100,000 person-years in individuals with 6, 4–5, 2–3 and 0–1 reference teeth respectively (Table 2). Among those with high levels of dental plaque the aSIR of MI was 731, 487 and 244 per 100,000 person-years for total, non-fatal and fatal MI respectively, compared to 388, 287 and 101 per 100,000 person-years (total, non-fatal and fatal MI respectively) for those with no dental plaque. An increased rate of total MI events was observed for all of the oral lesions (683, 675, 620 per 100,000 person-years for *Candida*-related, denture-related and tongue lesions, respectively) compared to the no lesions group (498 per 100,000 person-years) (Table 2). Similar increases in incidence rate with decreasing oral health status were observed when non-fatal and fatal MI events were analysed separately (Tables 3 and 4).

**Table 2:** Age-standardised incidence rates (aSIRs), hazard ratios (HRs) and 95 percent confidence intervals (95% CIs) of total (fatal and non-fatal) myocardial infarction (MI) by oral health status between 1973 and 2012.

	Cases of MI (N)	Person-years	aSIR (per 100,000 person-years)^*^	Minimally-adjusted HR ^†^(95% CI)	Fully-adjusted HR^‡^ (95% CI)
**Number of teeth** ^**§**^					
6	731	310,713	410.0	1.00	1.00
4–5	650	105,356	523.8	1.20 (1.07–1.35)	1.16 (1.04–1.31)
2–3	517	51,847	638.8	1.42 (1.25–1.61)	1.34 (1.17–1.52)
0–1	1,073	80,294	993.8	1.57 (1.39–1.77)	1.45 (1.28–1.64)
*P*-trend				<0.001	<0.001
**Dental plaque**					
No plaque	326	100,536	387.8	1.00	1.00
Low	1,096	298,161	467.3	1.05 (0.92–1.19)	1.04 (0.92–1.18)
High	476	69,219	731.2	1.36 (1.18–1.58)	1.32 (1.13–1.53)
Not accurate^||^	1,073	80,294	993.8	—	—
**Oral lesions**					
No lesions^#^	1,655	399,745	497.9	1.00	1.00
*Candida*-related	244	24,487	682.8	1.22 (1.06–1.40)	1.15 (1.00–1.33)
Denture-related	956	85,835	674.6	—	—
age <80 years^**^	572	35,385	1,488.6	1.29 (1.16–1.44)	1.22 (1.09–1.36)
age ≥80 years^**^	384	50,450	181.6	1.16 (1.00–1.34)	1.10 (0.95–1.29)
Tongue	572	74,578	620.3	1.16 (1.05–1.28)	1.16 (1.05–1.28)

^*^Age-standardised incidence rate per 100,000 person-years, standardised to the age distribution of person-years experienced by all participants, using 5-year age categories. ^†^Minimally-adjusted HRs: All Cox proportional hazards regression models had attained age as the time scale, stratified by sex (male, female) and attained calendar period in 5-year intervals. ^‡^Fully-adjusted HRs: All Cox proportional hazards regression models had attained age as time scale, adjusted for alcohol consumption (no/low, moderate/high) and area of residence (small town, rural, city), and stratified by sex (male, female), smoking and snus use (uses neither, smoker only, snus user only, uses both) and attained calendar period in 5-year intervals. ^§^Number of teeth was assessed at baseline through examination of six reference teeth (tooth numbers 16, 21, 24, 36, 41, 44 using the Ramfjord teeth index). Minimally-adjusted model (n = 20,133); Fully-adjusted model (n = 20,125). ^||^The measurement of dental plaque for individuals with only 0 or 1 of the reference teeth present was considered to not be accurate (n = 4,624). ^#^Reference group included those without any evidence of *Candida*-related, denture-related, or tongue lesions. ^**^Refers to attained age. The Cox regression model additionally contained an interaction term between attained age (<80 years, ≥80 years) and denture-related lesions, and was further stratified by area of residence in the fully-adjusted model but not in the minimally-adjusted model.

**Table 3:** Age-standardised incidence rates (aSIRs), hazard ratios (HRs) and 95 percent confidence intervals (95% CIs) of non-fatal myocardial infarction (MI) by oral health status between 1973 and 2012.

	Cases of MI (N)	Person-years	aSIR (per 100,000 person-years)^*^	Minimally-adjusted HR^†^ (95% CI)	Fully-adjusted HR^‡^ (95% CI)
**Number of teeth** ^**§**^					
6	609	310,713	314.6	1.00	1.00
4–5	459	105,356	368.3	1.13 (0.99–1.29)	1.10 (0.96–1.25)
2–3	333	51,847	423.2	1.30 (1.12–1.52)	1.24 (1.06–1.44)
0–1	633	80,294	727.9	1.43 (1.24–1.65)	1.32 (1.14–1.53)
*P*-trend				<0.001	<0.001
**Dental plaque**					
No plaque	248	100,536	286.7	1.00	1.00
Low	831	298,161	341.3	1.03 (0.89–1.19)	1.03 (0.89–1.19)
High	322	69,219	487.3	1.26 (1.06–1.49)	1.23 (1.03–1.47)
Not accurate^||^	633	80,294	727.9	—	—
**Oral lesions**					
No lesions^#^	1,189	399,745	348.1	1.00	1.00
*Candida*-related	145	24,487	434.8	1.17 (0.98–1.40)	1.10 (0.91–1.31)
Denture-related	590	85,835	439.2	1.25 (1.12–1.39)	1.20 (1.07–1.34)
Tongue	368	74,578	408.3	1.14 (1.01–1.28)	1.13 (1.00–1.28)

^*^Age-standardised incidence rate per 100,000 person-years, standardised to the age distribution of person-years experienced by all participants, using 5-year age categories. ^†^Minimally-adjusted HRs: All Cox proportional hazards regression models had attained age as the time scale, stratified by sex (male, female) and attained calendar period in 5-year intervals. ^‡^Fully-adjusted HRs: All Cox proportional hazards regression models had attained age as time scale, adjusted for alcohol consumption (no/low, moderate/high) and area of residence (small town, rural, city) and stratified by sex (male, female), smoking and snus use (uses neither, smoker only, snus user only, uses both) and attained calendar period in 5-year intervals. ^§^Number of teeth was assessed at baseline through examination of six reference teeth (tooth numbers 16, 21, 24, 36, 41, 44 using the Ramfjord teeth index). Minimally-adjusted model (n = 20,133); Fully-adjusted model (n = 20,125). ^||^The measurement of dental plaque for individuals with only 0 or 1 of the reference teeth present was considered to not be accurate (n = 4,624). ^#^Reference group included those without any evidence of *Candida*-related, denture-related, or tongue lesions.

**Table 4:** Age-standardised incidence rates (aSIRs), hazard ratios (HRs) and 95 percent confidence intervals (95% CIs) of fatal myocardial infarction (MI) by oral health status between 1973 and 2012.

	Cases of MI (N)	Person-years	aSIR (per 100,000 person-years)^*^	Minimally-adjusted HR^†^ (95% CI)	Fully-adjusted HR^‡^ (95% CI)
**Number of teeth§**					
6	122	310,713	95.3	1.00	1.00
4–5	191	105,356	155.5	—	—
age <80 years^||^	110	60,559	225.0	1.58 (1.16–2.14)	1.51 (1.11–2.05)
age ≥80 years^||^	81	44,796	69.8	1.31 (0.88–1.96)	1.28 (0.85–1.92)
2–3	184	51,847	215.6	—	—
age <80 years^||^	105	21,305	497.2	2.21 (1.60–3.05)	2.06 (1.49–2.84)
age ≥80 years^||^	79	30,542	77.7	1.32 (0.87–2.00)	1.25 (0.82–1.90)
0–1	440	80,294	265.9	—	—
age <80 years^||^	223	26,434	597.3	2.75 (2.03–3.74)	2.48 (1.82–3.37)
age ≥80 years^||^	217	53,861	86.3	1.30 (0.88–1.93)	1.29 (0.87–1.91)
*P*-trend (age <80)				<0.001	<0.001
*P*-trend (age ≥80)				0.38	0.40
**Dental plaque**					
No plaque	78	100,536	101.1	1.00	1.00
Low	265	298,161	126.0	1.10 (0.85–1.42)	1.07 (0.83–1.39)
High	154	69,219	244.0	1.66 (1.25–2.21)	1.56 (1.16–2.09)
Not accurate^#^	440	80,294	265.9	—	—
**Oral lesions**					
No lesions^**^	466	399,745	149.8	1.00	1.00
*Candida*-related	99	24,487	248.0	1.30 (1.03–1.63)	1.24 (0.99–1.57)
Denture-related	366	85,835	235.5	—	—
age <80 years^||^	192	35,385	519.9	1.46 (1.20–1.78)	1.37 (1.12–1.68)
age ≥80 years^||^	174	50,450	82.2	1.02 (0.82–1.26)	1.01 (0.81–1.26)
Tongue	204	74,578	212.0	1.20 (1.02–1.43)	1.20 (1.02–1.43)

^*^Age-standardised incidence rate per 100,000 person-years, standardised to the age distribution of person-years experienced by all participants, using 5-year age categories. ^†^Minimally-adjusted HRs: All Cox proportional hazards regression models had attained age as the time scale, stratified by sex (male, female) and attained calendar period in 5-year intervals. ^‡^Fully-adjusted HRs: Cox proportional hazards regression models had attained age as time scale, adjusted for alcohol consumption (no/low, moderate/high) and area of residence (small town, rural, city), and stratified by sex, smoking and snus use (uses neither, smoker only, snus user only, uses both) and attained calendar period in 5-year intervals. ^§^Number of teeth was assessed at baseline through examination of six reference teeth (tooth numbers 16, 21, 24, 36, 41, 44 using the Ramfjord teeth index). ^||^Refers to attained age. Model contained an interaction term between the oral health exposure and attained age (<80 years, ≥80 years). ^#^The measurement of dental plaque for individuals with only 0 or 1 of the reference teeth present was considered to not be accurate (n = 4,624). ^**^Reference group included those without any evidence of *Candida*-related, denture-related, or tongue lesions.

### Cox proportional hazards regression

A dose-response association was observed of increasing hazard for total MI events with decreasing number of teeth (*P*-value for trend < 0.001). Fully-adjusted HR was 1.16, 1.34 and 1.45 in individuals with 4–5, 2–3 and 0–1 reference teeth remaining respectively, compared to all 6 reference teeth remaining (Table 2). A similar increasing hazard was observed in the group of non-fatal MI events (Table 3). Results were separated into attained age <80 years and attained age ≥80 years in the analysis of fatal MI events, as the variable ‘number of teeth’ violated the proportional hazards assumption in this analysis. An increasing trend in hazard with decreasing number of teeth is only observed in the younger group (attained age <80 years), HR 1.51, 2.06 and 2.48 in individuals with 4–5, 2–3 and 0–1 reference teeth remaining respectively, compared to the group with all 6 reference teeth remaining (*P*-value for trend <0.001). In the group attained age ≥80 years, *P*-value for trend was 0.40 (Table 4).

High levels of dental plaque were associated with an increased hazard of MI events, compared to those with no dental plaque, in fully-adjusted models: HR 1.32 (95% CI 1.13 to 1.53), 1.23 (95% CI 1.03 to 1.47), 1.56 (95% CI 1.16 to 2.09) for total, non-fatal and fatal MI respectively (Tables 2–4).

Presence of *Candida*-related, denture-related or tongue lesions was associated with a small increased hazard of total, non-fatal and fatal MI events, of borderline significance (Tables 2–4). For total MI, HR was 1.15 (95% CI 1.00 to 1.33) for presence of *Candida*-related lesions; the corresponding estimate for denture-related lesions was 1.22 (95% CI 1.09 to 1.36) among those ages < 80 years; and the HR for presence of tongue lesions was 1.16 (95% CI 1.05 to 1.28), in fully-adjusted models (Table 2).

There was no significant interaction on the multiplicative scale between sex and any of the oral health variables (data not shown). However, in the Cox regression analyses conducted separately in males and females, the point estimates are generally larger in the female subgroup (Supplementary Tables S1, S2). Furthermore, there was no significant association between high dental plaque and total MI in females, HR 1.09 (95% CI 0.81 to 1.46) (Supplementary Table S1). In contrast, in the male-only analysis, a high level of dental plaque was significantly associated with total MI, HR 1.34 (95% CI 1.11 to 1.61) (Supplementary Table S2).

### Non-tobacco user subgroup analysis

The analysis was conducted separately in non-tobacco users only in order to minimise possible residual confounding by smoking and snus use. Among the non-tobacco users, the HR point estimates for total, non-fatal and fatal MI events were attenuated compared to the full cohort, but remained statistically significant for number of teeth lost for total and non-fatal MI (*P*-value for trend 0.007, 0.039 and 0.079 for total, non-fatal and fatal MI respectively) (Table 5). There were fewer cases of MI in the non-tobacco user subgroup compared to the full analysis and so less power to detect significant associations.

**Table 5:** Hazard ratios (HRs) and 95 percent confidence intervals (95% CIs) of myocardial infarction (MI) by oral health status between 1973 and 2012, among non-tobacco users only.

	Non-tobacco users					
	Total MI		Non-fatal MI		Fatal MI	
	Cases (N)	Adjusted HR^*^ (95% CI)	Cases (N)	Adjusted HR^*^ (95% CI)	Cases (N)	Adjusted HR^*^ (95% CI)
**Number of teeth** ^**†**^						
6	292	1.00	235	1.00	57	1.00
4–5	271	1.10 (0.92, 1.32)	187	1.09 (0.88, 1.34)	84	1.17 (0.81, 1.67)
2–3	205	1.15 (0.93, 1.41)	124	1.07 (0.83, 1.37)	81	1.33 (0.91, 1.93)
0–1	501	1.30 (1.07, 1.58)	297	1.29 (1.01, 1.63)	204	1.37 (0.95, 1.96)
*P*-trend		0.007		0.039		0.079
**Dental plaque**						
No plaque	164	1.00	121	1.00	43	1.00
Low	450	0.96 (0.80, 1.15)	329	0.93 (0.75, 1.15)	121	1.02 (0.71, 1.46)
High	154	1.12 (0.89, 1.41)	96	1.01 (0.76, 1.34)	58	1.40 (0.92, 2.13)
Not accurate^‡^	501	—	297	—	204	—
**Oral lesions**						
No lesions^§^	661	1.00	463	1.00	198	1.00
*Candida*-related	118	1.19 (0.96, 1.47)	66	1.15 (0.87, 1.52)	52	1.25 (0.90, 1.74)
Denture-related	448	1.06 (0.93, 1.21)	264	1.06 (0.89, 1.25)	184	1.06 (0.85, 1.32)
Tongue	252	1.02 (0.88, 1.19)	165	1.05 (0.87, 1.26)	87	0.97 (0.75, 1.26)

^*^Cox proportional hazards regression models had attained age as time scale, adjusted for alcohol consumption (no/low, moderate/high) and area of residence (small town, rural, city), and stratified by sex (male, female) and attained calendar period in 5-year intervals. ^†^Number of teeth was assessed at baseline through examination of six reference teeth (tooth numbers 16, 21, 24, 36, 41, 44 using the Ramfjord teeth index). ^‡^The measurement of dental plaque for individuals with only 0 or 1 of the reference teeth present was considered to not be accurate (n = 4,624). ^§^Reference group included those without any evidence of *Candida*-related, denture-related, or tongue lesions.

## Discussion

We hypothesised that poor oral health is associated with an increased risk of incident MI events. Our hypothesis was somewhat supported by the results of this study. In this population-based cohort study, we observed an increased risk of total, non-fatal and fatal MI events among individuals with fewer teeth, high levels of dental plaque and a borderline significant increased risk among individuals with oral lesions. Howell *et al*. suggested that a clinically significant association would be a 50% increased risk of CVD in those with poor oral health^31^. This study demonstrates a greater than 50% increased hazard of fatal MI events for all degrees of tooth loss (4–5, 2–3 and 0–1 teeth remaining compared to all 6 reference teeth remaining) in the younger age group (attained age < 80 years) and with high levels of dental plaque compared to no dental plaque. However, separate analysis in non-tobacco users showed an attenuation of some estimates to non-significance at the 0.05 significance level, indicating that the positive associations observed may be, at least in part, due to residual confounding by tobacco use.

Previous cohort and case-control studies have indicated a small but significant increased risk of MI among edentulous individuals^12^. A systematic review and meta-analysis study observed a similar increased risk of total and fatal coronary heart disease/CVD events with 0–10 teeth present at baseline compared to those with 25–32 teeth present^32^. The results from our analysis are in agreement with these studies. However, among non-tobacco users, a positive association is only observed in the group with 0–1 of the reference teeth present. One possible contributing explanation could be that those with the most tooth loss may have a poorer diet, resulting in an increased risk of MI. Studies in the US and Sweden have shown that tooth loss is associated with a diet low in fibre, fruit and vegetables but high in fat and sugar, which may be due to decreased masticatory ability^33,34^. Furthermore, increased risk of MI could be the result of prior periodontal disease, which has been associated with an increased risk of tooth loss and of a range of CVDs, through the proposed pathway of infection and inflammation causing atherosclerosis and thrombus formation^35^.

An increased risk of MI among individuals with high levels of dental plaque compared to those with no plaque was observed in this study. This is in line with a previous study which observed a positive association between death from heart infarction and dental calculus index^36^. High levels of dental plaque or calculus require a long time, often years, to develop and therefore indicates prolonged poor oral health. Dental plaque is a biofilm of bacteria located at the gingival margin of the teeth and gums which can be a source of chronic inflammation. However, analysis in this cohort of only non-tobacco users showed no significant association between dental plaque and total, non-fatal or fatal MI events, which indicates that perhaps residual confounding by tobacco use is responsible for the positive association observed in the full cohort.

To the best of our knowledge, no previous longitudinal studies have investigated the association between a range of different oral lesions and risk of MI. A previous cross-sectional study indicated an association between various oral lesions and increased risk of cardiovascular diseases while controlling for various potential confounding factors^37^. In this study, we observed a borderline significant increased risk of MI with various oral lesions. As with other measures of oral health in this study, the HR for the presence of denture-related lesions was greater in the younger attained age groups (<80 years). This could be because old individuals with the greatest likelihood of death due to MI had already died before entry into the study; if these individuals also had the poorest oral health then this could result in an underestimation of the HR in the older age group. In the analysis among non-tobacco users, the point estimates are non-significant.

In all analyses, the minimally-adjusted and fully-adjusted HRs in this study are similar. This is in accordance with other studies which observed little change in the point estimates when other coronary heart disease risk factors, such as hypertension and hypercholesterolemia, and dietary factors, were added to the regression models^12^.

There have been hypotheses for a causal effect of poor oral health on the risk of MI. Presence of pathogenic bacteria in the mouth, such as *Porphyromonas gingivalis*, can result in inflammation. Inflammation and the systemic release of inflammatory mediators such as C-reactive protein (CRP) are suggested to be associated with an increased risk of CVD development^38,39^. Over 50 different pathogenic and commensal oral bacteria have been identified in atherosclerotic plaques, and the abundance of the bacteria identified in the plaque correlates with the species of bacteria present in the mouth of the individual, suggesting that the oral cavity is one of the sites of entry of bacteria into the blood^40^.

There are some limitations of the study. There is likely to be uncontrolled confounding as there is no information regarding diet, medication use, genetic polymorphisms^41^ and BMI, all of which are suggested risk factors for both poor oral health and MI. Diabetes mellitus is also a potential confounding factor however information on diabetes and other comorbidities was not collected at study entry. In the cohort (n = 20,133) only 204 individuals (1.0%) had a record of diabetes in the Patient Register before entry into the cohort which indicates underestimation of diabetes using the Patient Register. Therefore, diabetes was not included in the Cox proportional hazards regression models. Uncontrolled confounding could result in overestimation of the HRs. Place of residence is the only indicator of socioeconomic status (SES) which will not capture all of the variation in SES, thus there is likely to be residual confounding which would most likely result in overestimation of the HRs. The exposures and other covariate values were only measured once at baseline and it is likely that there has been a change in the values of some of these variables during the long follow-up period. The most likely situation is that people will develop poorer oral health as they age, as poor oral health is strongly associated with increasing age^42^. The effect of this misclassification of the exposure would most likely act to shift the estimate towards the null, and therefore is unlikely to explain the associations observed in this study. An advantage of using only baseline measurements is the reduced likelihood of changes in health-related behaviours due to the outcome, for example, being diagnosed as at high risk of developing MI may lead to some individuals reducing their tobacco and alcohol consumption.

There is likely to be underreporting of alcohol consumption as this information is self-reported. Only 60 of the 20,133 individuals (0.3%) reported high (daily) alcohol consumption. In contrast a recent study of community-dwelling individuals aged 65–95 years in Copenhagen, Denmark observed a prevalence of daily alcohol consumption of 30%^43^. In order to reduce the chance of misclassification, moderate and high alcohol consumption in this study was combined into a single group. This could be a problem as some studies have suggested a J-shaped association between alcohol and CVD risk, with moderate levels being protective against CVD and high levels being a risk factor^44^. Nevertheless, this association is disputed and a recent paper using a Mendelian randomisation study design, which limits uncontrolled confounding bias, concluded that reducing alcohol consumption is beneficial for cardiovascular health, even among moderate alcohol consumers^45^.

There are also some advantages of this study. Measurement of oral health is likely to be accurate in this study as it was assessed by a dentist, rather than by self-report which is often used in large epidemiological studies. The same dentist (T. Axéll) performed all the dental examinations, thus removing inter-examiner differences. The risk of selection bias is low due to the use of the Swedish health and population registers, with very few lost to follow-up. The fact that the cohort was not developed with the current study hypothesis in mind ensured that diagnosis of oral health conditions was not affected by factors related to MI. There were few missing values for covariates: missing smoking and snus use (n = 2), missing alcohol consumption (n = 9), therefore very few individuals were excluded when performing analyses using the fully-adjusted Cox proportional hazards models. The use of the National Registers also reduces the risk of detection bias, as data about MI events was collected in the same way in exposed and unexposed individuals. Other advantages of the study include high statistical power (total number of MI cases = 2,971), and long follow-up period (1973–2012), which covers the postulated 10-year induction period of CHD resulting from chronic inflammation^8^. A meta-analysis by Humpreys *et al*.^32^, observed a significantly higher risk ratio for the association between periodontitis and CHD/CVD in studies with a follow-up period of greater than 15 years, compared to a follow-up time of 15 years or less. The varying length of follow-up in different studies could be partly responsible for the inconsistent results observed.

Large numbers of people are affected by poor oral health and MI, meaning that understanding the relationship between oral health and MI could provide multiple advantages to public health. This study may have good generalisability to other populations, at least within Sweden and perhaps also other countries in Northern Europe as the cohort is likely to be representative in terms of oral health, age, socioeconomic status and health behaviours such as tobacco use and alcohol consumption.

In summary, in this large population-based cohort study, tooth loss, presence of high levels of dental plaque and presence of oral lesions, are associated with a slightly increased hazard of MI events, even after controlling for potential confounders. However, these results may be partly explained by residual confounding. Thus, although it may be important to consider oral hygiene interventions to maintain good overall health, this is unlikely to be effective for greatly reducing the risk of MI, unless individuals also reduce other risk factors for MI such as to stop smoking.

## Electronic supplementary material

Supplementary tables
