# Supplementary material for: Poor oral health and risk of incident myocardial infarction: A prospective cohort study of Swedish adults, 1973–2012
Source: Sci Rep. 2018 Jul 31;8:11479. doi: 10.1038/s41598-018-29697-9 (PMC6068156; doi:10.1038/s41598-018-29697-9)
Supplement: Supplementary file 1 — Supplementary tables [file 41598_2018_29697_MOESM1_ESM.pdf]

Supplementary tables.

Poor oral health and risk of incident myocardial infarction: A prospective cohort study of Swedish adults, 1973-2012.

Katherine Wilson[1], Zhiwei Liu[1], Jiaqi Huang[1], Ann Roosaar[2], Tony Axéll[3], Weimin Ye[1*]

[1]Department of Medical Epidemiology and Biostatistics, Karolinska Institutet, Stockholm, Sweden;

[2] Department of Dental Medicine, Karolinska Institutet, Stockholm, Sweden;

[3] Maxillofacial Unit, Halmstad Hospital Halland, 30185 Halmstad, Sweden.

Supplementary Table S1. Hazard ratios (HRs) and 95% confidence intervals (CIs) of total (fatal and non-fatal) myocardial infarction (MI) by oral health status between 1973 and 2012 among females only (n=10,218).

	Cases of MI (N)	Person-years	Adjusted HR* (95% CI)
**Number of teeth†**			
6	175	153,372	1.00
4-5	227	56,922	--
age <80 years‡	148	31,474	1.31 (1.02 – 1.69)
age ≥80 years‡	79	25,448	1.16 (0.77 – 1.76)
2-3	183	28,414	--
age <80 years‡	102	10,202	1.46 (1.09 – 1.95)
age ≥80 years‡	81	18,212	1.24 (0.81 – 1.88)
0-1	537	50,378	--
age <80 years‡	264	14,499	1.80 (1.38 – 2.34)
age ≥80 years‡	273	35,879	1.31 (0.88 – 1.94)
**Dental plaque**			
No plaque	158	68,642	1.00
Low	359	149,594	1.11 (0.92 – 1.35)
High	67	20,442	1.09 (0.81 – 1.46)
**Oral lesions**			
No lesions§	520	205,114	1.00
Candida-related	117	13,599	1.20 (0.97 – 1.49)
Denture-related	485	53,254	1.21 (1.05 – 1.39)
Tongue	214	38,255	1.16 (0.98 – 1.37)

* Adjusted HR: Cox proportional hazards regression model with attained age as the time scale, adjusted for alcohol consumption (no/low, moderate/high) and area of residence (small town, rural, city), and stratified by smoking and snus use (uses neither, smoker only, snus user only, uses both) and attained calendar period in 5-year intervals. Denture-related lesions and number of teeth models were further stratified by area of residence.

† Number of teeth was assessed at baseline through examination of six reference teeth (tooth numbers 16, 21, 24, 36, 41, 44 using the Ramfjord teeth index).

‡ Refers to attained age. Model contained an interaction term between the oral health exposure and attained age (<80 years, ≥80 years).

§ Reference group included those without any evidence of *Candida*-related, denture-related, or tongue lesions.

Supplementary Table S2. Hazard ratios (HRs) and 95% confidence intervals (CIs) of total (fatal and non-fatal) myocardial infarction (MI) by oral health status between 1973 and 2012 among males only (n=9,915).

	Cases of MI (N)	Person-years	Adjusted HR* (95% CI)
**Number of teeth†**			
6	556	157,332	1.00
4-5	423	8,739	1.13 (0.98 – 1.30)
2-3	333	14,231	1.34 (1.15 – 1.57)
0-1	536	17,976	1.37 (1.18 – 1.60)
**Dental plaque**			
No plaque	167	31,843	1.00
Low	737	148,537	0.99 (0.84 – 1.18)
High	408	48,755	1.34 (1.11 – 1.61)
**Oral lesions**			
No lesions‡	1,135	194,478	1.00
Candida-related	127	10,885	1.12 (0.92 – 1.35)
Denture-related	470	32,510	1.17 (1.04 – 1.31)
Tongue	357	36,298	1.16 (1.02 – 1.31)

* Adjusted HR: Cox proportional hazards regression model with attained age as the time scale, adjusted for alcohol consumption (no/low, moderate/high) and area of residence (small town, rural, city), and stratified by smoking and snus use (uses neither, smoker only, snus user only, uses both) and attained calendar period in 5-year intervals.

† Number of teeth was assessed at baseline through examination of six reference teeth (tooth numbers 16, 21, 24, 36, 41, 44 using the Ramfjord teeth index).

‡ Reference group included those without any evidence of *Candida*-related, denture-related, or tongue lesions.
